# Dietary Trace Elements and the Pathogenesis of Neurodegenerative Diseases

**DOI:** 10.3390/nu15092067

**Published:** 2023-04-25

**Authors:** Masahiro Kawahara, Midori Kato-Negishi, Ken-ichiro Tanaka

**Affiliations:** Department of Bio-Analytical Chemistry, Research Institute of Pharmaceutical Sciences, Faculty of Pharmacy, Musashino University, Tokyo 202-8585, Japan; mnegishi@musashino-u.ac.jp (M.K.-N.); k-tana@musashino-u.ac.jp (K.-i.T.)

**Keywords:** amyloid, neurotoxicity, conformation, reactive oxygen species (ROS), gastrointestinal absorption

## Abstract

Trace elements such as iron (Fe), zinc (Zn), copper (Cu), and manganese (Mn) are absorbed from food via the gastrointestinal tract, transported into the brain, and play central roles in normal brain functions. An excess of these trace elements often produces reactive oxygen species and damages the brain. Moreover, increasing evidence suggests that the dyshomeostasis of these metals is involved in the pathogenesis of neurodegenerative diseases, including Alzheimer’s disease, prion diseases, and Lewy body diseases. The disease-related amyloidogenic proteins can regulate metal homeostasis at the synapses, and thus loss of the protective functions of these amyloidogenic proteins causes neurodegeneration. Meanwhile, metal-induced conformational changes of the amyloidogenic proteins contribute to enhancing their neurotoxicity. Moreover, excess Zn and Cu play central roles in the pathogenesis of vascular-type senile dementia. Here, we present an overview of the intake, absorption, and transport of four essential elements (Fe, Zn, Cu, Mn) and one non-essential element (aluminum: Al) in food and their connections with the pathogenesis of neurodegenerative diseases based on metal–protein, and metal–metal cross-talk.

## 1. Introduction

Several essential trace elements, such as iron (Fe), zinc (Zn), copper (Cu), and manganese (Mn), are distributed in various regions of the brain [1]. These elements, termed “neurometals,” play important roles in various brain functions such as myelination, information processing of neural networks, neurotransmitter synthesis, memory formation, and protection of neural cells. These micronutrients are naturally obtained from food or drinking water and can be absorbed via the gastrointestinal tract. However, their absorption rates are generally low and are influenced by the chemical speciation of metal ions and by coexisting substances in food. Furthermore, the blood–brain barrier (BBB) protects the entrance of trace elements into the brain. Therefore, deficiency of these neurometals tends to occur, which has various adverse effects on normal brain functions, particularly in the developing brain.

An excess of these essential elements is also toxic. The oxidative stress and reactive oxygen species (ROS) cause damage to the brain because it uses about 20% of the total oxygen used in the body, despite its small volume (comprising around 2% of the body) [2]. Excess Fe and Cu, both redox-active metals, produce ROS and damage neurons. Excess Mn also induces oxidative stress and causes neurotoxicity, termed “manganism,” the symptoms of which are similar to Parkinson’s disease [3]. Excess Zn and Cu play key roles in neuronal death after transient global ischemia [4].

It is widely accepted that the disruption of homeostasis of these neurometals (excess as well as deficiency) is involved in the pathogenesis of various neurodegenerative diseases, including Alzheimer’s disease (AD), prion diseases, Lewy body diseases (LBD), and vascular-type senile dementia (VD) [5,6,7,8]. Among these, AD, prion diseases, and LBD share similar pathological hallmarks involving the deposition of disease-related proteins [AßP in AD, prion protein (PrP) in prion diseases, α-synuclein in LBD] in the patient’s brain. Although these proteins have completely different primary sequences, they share the characteristics of being involved in the formation of amyloid fibrils with ß-pleated sheet structures and exhibiting neurotoxicity. Thus, these neurodegenerative diseases are categorized as “conformational diseases” (protein misfolding diseases) [9], and these disease-related proteins are called “amyloidogenic proteins.”

The degenerative processes of these diseases implicate at least two possible pathogenic pathways: the first promotes the loss of normal, protective functions, while the second promotes the gain of toxic functions. Interestingly, trace elements are involved in both pathways. Regarding the first pathway, all of these amyloidogenic proteins commonly possess the ability to bind metals and regulate metal homeostasis [10]. As an additional commonality, they all co-localize at the synapses, which are the sites initially affected in the early stage of these diseases and are enriched in metals. Meanwhile, metal-induced conformational changes of amyloidogenic proteins enhance their neurotoxicity and contribute to the second pathway.

Furthermore, the interactions between metal ions at the synapse may also contribute to the pathogenesis of these neurodegenerative diseases. Different metal ions usually share binding sites of the same protein with different binding constants. For example, Cu^2+^ and Zn^2+^ often bind the same metal-binding proteins and interact with one another. Meanwhile, Mn^3+^, as well as aluminum (Al^3+^), share similar chemical characteristics with Fe^3+^ and are transported by binding with transferrin (TF), an iron-binding protein [11,12]. Several types of Zn transporters also transport Fe and Mn in addition to Zn [13]. Therefore, the cross-talk between metal ions at the synapses may also influence the pathogenesis of these neurodegenerative diseases. Moreover, it is known that Zn^2+^ can enter into neurons via voltage-dependent calcium channels (VDCC) and other Ca^2+^-permeable channels of glutamate receptors during neuronal depolarization [8]. Thus, the interaction between Ca^2+^ and other cations is crucial.

In this review, we focus on four essential elements (Fe, Zn, Cu, and Mn) and one toxic element (Al) and present an overview of their biological fates (such as the intake from food, absorption in the gastrointestinal tract, transport in the body, and entry into the brain) and their roles in maintaining normal brain functions. Thereafter, we review the involvement of these trace elements in the pathogenesis of neurodegenerative diseases from the perspectives of metal–protein, and metal–metal cross-talk. We also discuss possible treatments for neurodegenerative diseases based on metallomics studies.

## 2. Characteristics of Trace Elements and Their Roles in Biological Functions

### 2.1. Iron

Fe is the most abundant trace element in the brain as well as in the whole body [14]. The human body contains around 3–5 g of Fe, and approximately 70% of the body’s total Fe is contained in heme proteins such as hemoglobin and myoglobin. In the brain, Fe particularly accumulates in the globus pallidus, putamen, and substantia nigra, among others. The highest concentration of Fe in the brain corresponds to the level in the liver. Fe functions as a cofactor or a component of enzymes or functional proteins, including cytochrome C oxidase, catalase, cytochrome P-450, tyrosine hydroxylase, and succinate dehydrogenase. Fe is essential for numerous biological functions such as oxygen transport, mitochondrial respiration, DNA synthesis, oxidative phosphorylation, energy production, metabolism, myelin synthesis, and the synthesis of neurotransmitters (dopamine, catecholamine, serotonin). Therefore, Fe deficiency causes anemia, loss of the ability to exercise, and decreased intellectual ability [15]. Fe supplementation reportedly improves the academic test scores and working memory of adolescent women [16], along with the exercise ability of female athletes [17].

Excess Fe is also toxic. Fe forms ferric iron (Fe^3+^) and ferrous iron (Fe^2+^), which in turn produce ROS via the Fenton reaction. The chemical speciation of Fe is regulated by ferroxidases, which oxidize Fe^2+^ ions to Fe^3+^, and ferrireductases, reducing Fe^3+^ ions to Fe^2+^. Ferroptosis, a non-apoptotic form of programmed cell death caused by excess Fe, is associated with the pathogenesis of various diseases [18,19].

Dietary Fe exists principally as heme Fe and non-heme Fe [20]. Heme Fe is derived predominantly from hemoglobin and myoglobin in meat and is absorbed via transporters such as the heme carrier protein 1 (HCP-1) and a folate transporter with a relatively high absorption rate (10–20%). Conversely, non-heme Fe, largely found as Fe^3+^, is derived from vegetables and beans and is highly insoluble. Non-heme Fe^3+^ in such food is reduced to Fe^2+^ by a ferrireductase such as duodenal cytochrome b or by ascorbic acid in food. It is subsequently absorbed by the divalent metal transporter 1 (DMT1). The absorption rate of non-heme Fe is low (2–5%) and influenced by numerous dietary components. Dietary components commonly found in plants, such as phytate, oxalate, and polyphenols, decrease the absorption of non-heme Fe. Other metal ions, including Zn^2+^ and Cu^2+^, also use DMT1 and influence the absorption of Fe.

**Figure 1:**
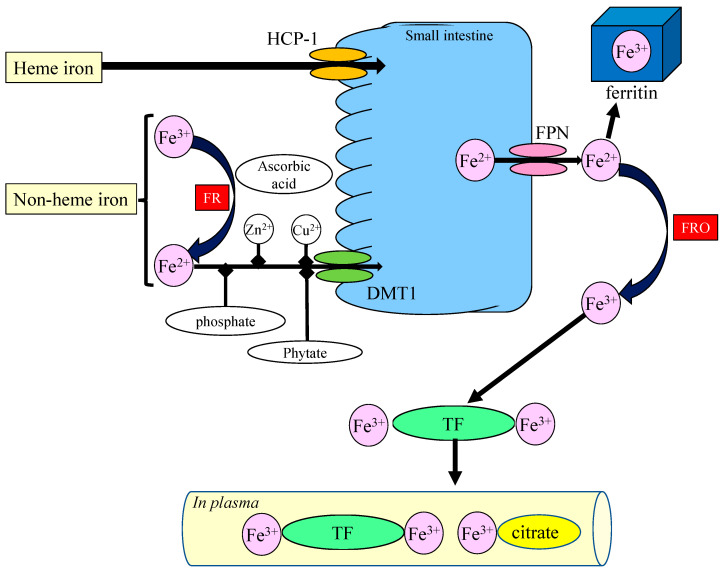
Intake, absorption, and transportation of iron in the body. Orally administered heme-Fe is absorbed via heme carrier protein (HCP-1) in the small intestine. Non-heme Fe is primarily in the form of Fe^3+^ and is reduced to Fe^2+^ by ferrireductase (FR), such as duodenal cytochrome b, or by ascorbic acid in food. Thereafter, Fe^2+^ is transported via divalent metal transporter (DMT1). The absorption is influenced by phosphate, phytate, Zn^2+^, and Cu^2+^ in food. Some of the absorbed Fe^2+^ is transported to the liver and stored in ferritin as Fe^3+^ or exported via ferroportin (FPN). The Fe^2+^ is oxidized to Fe^3+^ by ferroxidase (FRO), such as ceruloplasmin, binds to transferrin (TF) or citrate in the blood, and is transported.

Absorbed Fe^2+^ is oxidized and stored in ferritin, an iron-storage protein, or is exported to extracellular space via ferroportin (FPN), the Fe^2+^ exporter protein. Hepcidin, a peptide hormone, controls FPN and contributes to Fe metabolism [21]. The ferroxidases, such as ferritin or ceruloplasmin, convert Fe^2+^ ions to Fe^3+^, and the Fe^3+^ is transported in the blood flow by binding to TF, an iron-binding protein that binds two Fe^3+^ ions. TF-bound Fe crosses the BBB binding with TF receptors and enters neurons or glial cells. Other Fe [non-transferrin-bound iron (NBTI)] largely forms Fe^3+^ ions and binds to small molecules such as citrates, after which it is reduced to Fe^2+^ by ferrireductases, and enters neurons or glial cells via DMT1. DMT1 is also localized in endothelial cells of the BBB and functions in the translocation of Fe^2+^ through the BBB [22]. Intracellular Fe^2+^ is transferred to neuronal enzymes in the mitochondria, endoplasmic reticulum (ER), or Golgi apparatus, which require bioactive Fe^2+^ as a cofactor. FPN exports excess Fe^2+^ to the extracellular space and regulates its intracellular levels because excess Fe^2+^ causes ROS and damages neurons. Therefore, the ratio between Fe^2+^ and Fe^3+^, as well as the level of Fe, is strictly regulated. The complicated biological fate of Fe is illustrated in Figure 1.

Fe regulates the expression of various Fe-related proteins, such as ferritin, TF, FPN, and DMT1 [23]. The mRNA of these proteins possesses an iron-responsive element (IRE) in the 3′-UTR or 5′-UTR. For example, the mRNA of ferritin has the IRE motif in its 5′-UTR. In Fe-deficient conditions, iron-binding proteins (IRPs; IRP1 and IRP2) bind to the IRE motif and inhibit ferritin expression, which eventually promotes the circulation of Fe in the blood. Under conditions with an excess of Fe, Fe binds to IRPs, promotes their dissociation from ferritin’s mRNA, and enhances the translation of ferritin, which finally decreases the concentration of free Fe. In contrast, the IRE motif at the 3′-UTR of transferrin’s mRNA binds to IRPs in Fe-deficient conditions, protects mRNAs from endonuclease cleavage, and promotes the translation of transferrin and the circulation of Fe. This IRE-IRP signaling pathway controls the level of Fe in the brain as well as other organs. Since the mRNAs encoding amyloidogenic proteins also possess the IRE motif, this IRE-IRP pathway is also implicated in the pathogenesis of neurodegenerative diseases (see Section 3.1).

Owing to the low absorption rate of Fe, Fe deficiency is a common and serious problem for human health, especially in developing countries where meat consumption is insufficient. The World Health Organization (WHO) estimated that approximately 4–5.5 billion people globally have Fe deficiency, while two billion people exhibit Fe-deficient anemia [24]. According to the National Health and Nutrition Survey Japan 2019, the recommended daily intake of Fe is 6.5 mg for women and 7.5 mg for men [25]. However, the estimated average Fe intake is 7 mg per day, and around 13.5% of women and 10.0% of men suffer from Fe deficiency with decreased hemoglobin content. Meanwhile, excess Fe as a result of genetic disorders or the overdose of Fe causes hemochromatosis with the accumulation of Fe [26].

### 2.2. Zinc

Zn is the second most abundantly present in the brain [27]. A high concentration of Zn is observed in the hippocampus, amygdala, cerebral cortex, thalamus, and olfactory cortex. Zn plays important roles in various physiological functions such as mitotic cell division, protein synthesis, immunological responses, and DNA and RNA synthesis as a cofactor of more than 300 enzymes or metalloproteins. Zn is also responsible for the expression of various genes since many transcription factors possess Zn-finger domains. Zn acts similarly to a signaling molecule to facilitate the transcription of the signaling cascades, similar to Ca. Therefore, Zn is essential for normal growth and development. Prasad et al. reported cases of Zn deficiency in humans for the first time in 1961; this condition is characterized by dwarfism with the retardation of physical and sexual development [28]. Zn deficiency in children is also associated with delayed mental and physical development, immune dysfunction, and learning disabilities, whereas Zn deficiency in adults produces learning, taste, and odor disorders [29,30].

In the brain, a substantial fraction (approximately 10% or more) of Zn is stored as a complex with glutamate in the presynaptic vesicles in excitatory glutamatergic neurons (gluzinergic neurons). Synaptic Zn is secreted into synaptic clefts with glutamate during neuronal excitation as free Zn ions (Zn^2+^) or is in a loosely bound form [27,31]. The secreted Zn binds to various receptors such as *N*-methyl-D-aspartate (NMDA)-type glutamate receptors, α-amino-3-hydroxy-5-methylisoxazole-4-propionic acid (AMPA)-type glutamate receptors, γ-aminobutyric acid (GABA) receptors, and glycine receptors, and regulate their functions. Thus, the removal of synaptic Zn by its chelator was reported to induce overexcitation in rat hippocampal neurons. Zn released from mossy fibers in the hippocampus reportedly mediates the spatiotemporal information of neuronal signaling and contributes to synaptic plasticity [32]. Synaptically released Zn is required for the induction of long-term potentiation, a form of synaptic information storage well known as a paradigm of the mechanism by which memory is formed [33].

The maintenance of Zn homeostasis is regulated by two factors: metallothioneins (MTs) and Zn transporters. There are three types of MTs: MT1, MT2, and MT3. MTs are ubiquitous proteins with 20 cysteine residues that can bind seven atoms of Zn, Cu, Cd, and other metals [34]. Among them, MT3 is primarily localized in the central nervous system, while MT1 and MT2 are ubiquitously expressed throughout the body. Meanwhile, there are two types of Zn transporters: ZnT transporters and Zrt-, Irt-like protein (ZIP) transporters that regulate Zn efflux and influx [35]. ZnT1, one of the ZnT transporters that facilitate the efflux of Zn^2+^, plays central role in Zn^2+^ efflux in the brain. ZnT3 regulates Zn^2+^ into synaptic vesicles in membranes of synaptic vesicles. ZIP transporters control Zn influx into cells or subcellular organelles. Genetic dysregulation of these transporters causes Zn deficiency and leads to several diseases, such as Ehlers–Danlos syndrome in humans.

Zn is abundant in food such as meat, oysters, eggs, and liver. Zn in food is absorbed in the gastrointestinal tract via ZIP4 and DMT1, after which it is transported in the blood by binding with albumin and α1-acid glycoprotein. Although Zn is also contained in vegetables, beans, and grains, its absorption rate from these sources is low because phytate in such plants binds to Zn and inhibits its absorption. The recommended daily intake of Zn is around 8 mg for women, 25 mg for pregnant women, and 11 mg for men [25]. The WHO estimated that 2.2 billion people are affected by Zn deficiency and that Zn deficiency is believed to make a major contribution to infant mortality [36]. In Japan, studies have reported that 37.9% of elderly people aged over 60 years and 16.9% of people aged 15–59 years exhibited low serum Zn levels [37].

### 2.3. Copper

Cu is the third most abundant element in the brain. Cu is a cofactor for numerous enzymes, including cytochrome C, Cu/Zn superoxide dismutase (Cu/Zn-SOD), lysyl oxidase, and tyrosinase. Therefore, Cu is involved in normal brain functions such as neurotransmitter synthesis, myelination, and neuroprotection [38]. Cu is also involved in Fe homeostasis since ceruloplasmin and other ferroxidases, termed ‘multi-copper ferroxidases,’ convert Fe^2+^ to Fe^3+^ [39]. Similar to Zn^2+^, a considerable amount of Cu is stored in synaptic vesicles. The synaptic Cu is released into synaptic clefts during neuronal firings [40]. The released Cu^2+^ modulates neuronal excitability by binding to NMDA-type glutamate receptors, AMPA-type glutamate receptors, and GABA receptors [41]. The concentrations of Cu and Zn in the synaptic clefts are considered to be higher compared to those in CSF, and Zn as well as Cu will play critical roles in the neurodegenerative processes (see Section 4). Meanwhile, excess free Cu is toxic because Cu exists as oxidized Cu^2+^ and reduced Cu^+^ and produces ROS.

Cu homeostasis is regulated by copper-transporting ATPase (ATP7A and ATP7B). A deficiency or excess of Cu caused by genetic disorders associated with these transporters can cause neurodegenerative diseases such as Menkes disease and Wilson’s disease [42]. ATP7A is a Cu efflux protein, delivering Cu to Cu-containing enzymes in the trans-Golgi network. ATP7A is mainly localized in the Golgi apparatus and reportedly plays an essential role in axonal targeting and synaptogenesis [43]. ATP7B has functional similarity to ATP7A and exports Cu into bile for excretion. Excess intracellular Cu can bind to MTs, as well as Zn. Cu transporter 1 (Ctr1), which transports Cu^+^, and DMT1, also contribute to Cu homeostasis.

Cu is abundant in meat, cereals, vegetables, and seafood such as squid and octopus. Orally ingested Cu is absorbed via DMT1 in the small intestine, mainly in the duodenum, transported to the liver, and distributed by ceruloplasmin in the blood. The daily intake of Cu is estimated to be 0.7 mg. Thus, Cu deficiency is rare in physiological conditions. Meanwhile, excess Cu in drinking water is associated with non-Indian childhood cirrhosis, a form of early childhood liver cirrhosis [44].

### 2.4. Manganese

Mn acts as a cofactor of various enzymes, such as hydrolases, lyases, and ligases, and is involved in metabolic processes, such as protein glycosylation and lipid synthesis. Mn also participates as a cofactor of superoxide dismutase (Mn-SOD), which detoxifies free radicals [45] and influences neurotransmitter synthesis as a component of glutamine synthetase.

Mn is contained in seaweed, nuts, and other food [46]. Orally administered Mn becomes Mn^2+^ in the presence of gastric acid, which is then oxidized to Mn^3+^. This Mn^3+^ is transported by binding to TF or citrate and can enter the brain via TF receptors, citrate transporters, and other transporters such as DMT1. Some Zn transporters, such as ZnT10, ZIP8, and ZIP14, control the absorption of Mn [13]. The recommended intake of Mn is 4 mg for men and 3.5 mg for women in Japan [23].

Mn deficiency is rare in physiological conditions because Mn is present at adequate levels in food. Mn is also used in fuel additives or fungicides and enters the body via the inhalation of fumes, dust, or air particles [47]. Excess Mn from contamination or occupational exposure is toxic and causes neurotoxicity called “manganism,” which involves symptoms similar to those of Parkinson’s disease (PD) [48]. Mn also reportedly causes oxidative stress and inflammatory responses. Since Mn has similar chemical characteristics to Fe, it influences Fe homeostasis and expressions of Fe-related proteins by affecting the IRE-IRP pathways [49].

### 2.5. Aluminum

Al is the third most abundant element in the earth’s crust and is widely distributed throughout the environment. Al has been widely used as compounds such as clay, glass, and alum for centuries. However, Al as a silvery metal was first isolated in 1827, and its industrial use began only in 1886.

Despite being widespread in the environment, Al is not essential for life. However, Al is known to inhibit more than 300 biologically significant reactions [50,51]. Al tightly binds to phosphate and inhibits kinases or phosphatases. The ligand-exchange rate for Mg^2+^ is 10^5^ times faster than that for Al^3+^, whereas the exchange rate for Al^3+^ is 10^8^ times slower than that for Ca^2+^. Therefore, Al^3+^ cannot participate in Ca^2+^- or Mg^2+^-related enzymatic reactions but inhibits numerous Ca^2+^- or Mg^2+^-related enzymes. Al^3+^ has similar characteristics to Fe^3+^, and thus Al^3+^ can bind to Fe-binding proteins or chelators. Although Al^3+^ is not redox-active, Al is known to enhance Fe-induced lipid peroxidation.

Al is naturally contained in several types of food, such as seaweed and tea leaves. Al can also enter the human body through food additives (such as alum and baking powder), contamination from cooking utensils, and drinking water because Al coagulants are widely used in water purification. Al has been used as a pharmacological agent, such as antacids, antiperspirants, and vaccines. Our daily intake of Al is estimated to be approximately 20 mg. However, its rate of absorption from the gastrointestinal tract is low and can be altered by its particular chemical form and substances coexisting in food [52]. For example, Al in tea infusion is poorly absorbed since polyphenol inhibits its absorption. Analysis using a non-radioactive isotope of Al (^26^Al) revealed that orally administered Al could be absorbed and accumulate in bones, and a considerable amount of Al can enter the brain [53]. Moreover, its biological half-life is quite long, and the level of Al in the brain is not changed after one month of administration [54].

Owing to these adverse characteristics, Al is a neurotoxin. Al causes dialysis encephalopathy in hemodialysis patients since Al is used as a component of hemodialysis solutions or pharmacological compounds. Al is also eluted from the soil by acid rain and is toxic to plants and fish. In addition, numerous studies have suggested its causative links between various neurodegenerative disorders, including AD, amyotrophic lateral sclerosis in the Kii Peninsula and Guam, Gulf War syndrome, and autism spectrum disorder [51]. In particular, the link between Al and AD has been debated for decades but remains controversial.

## 3. Roles of Trace Elements in the Pathogenesis of Neurodegenerative Diseases

### 3.1. Alzheimer’s Disease

Growing evidence suggests the involvement of trace elements in the pathogenesis of neurodegenerative diseases such as AD, prion diseases, LBD, and VD.

AD is responsible for approximately half of all cases of senile-type dementia. The hallmarks of senile-type dementia are profound memory loss and an inability to form new memories. The age-dependent increase in its prevalence is a serious problem in the aging world. The number of AD patients in Japan was estimated to be more than 7 million in 2020, a number that is increasing annually [55]. The hallmarks of AD are the loss of neurons and synapses in the hippocampus or cerebral cortex, the extracellular accumulation of senile plaques, and the intraneuronal accumulation of neurofibrillary tangles (NFTs) [56]. The major constituent of senile plaques is AßP, while that of NFTs is phosphorylated tau protein.

**Figure 2:**
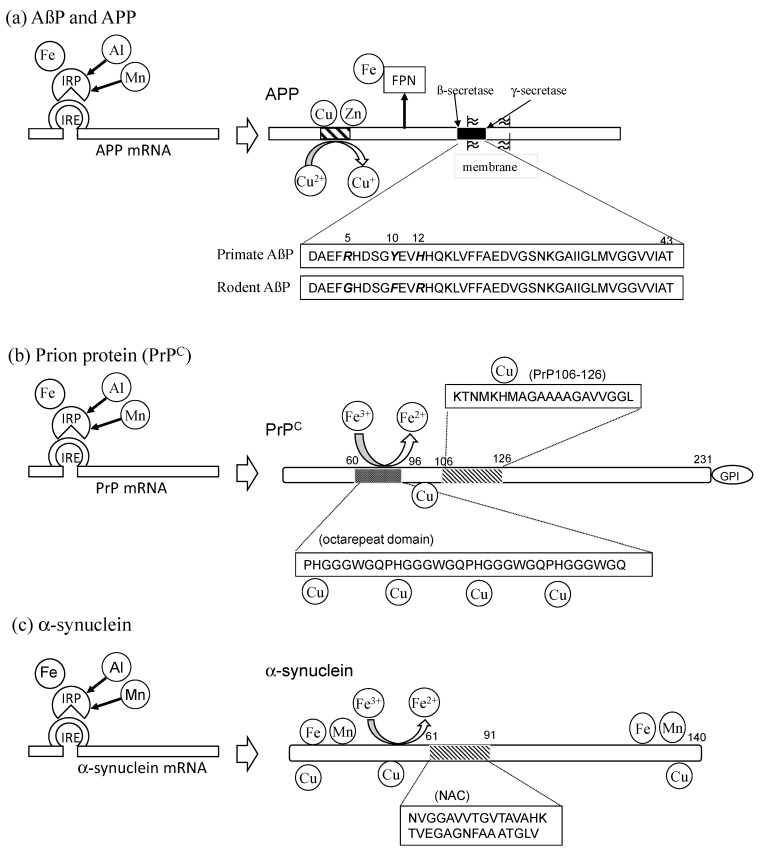
Characteristics of amyloidogenic proteins and the link with trace elements. (**a**) Amyloid precursor protein (APP) and ß-amyloid protein (AßP). The expression of APP is regulated by the level of Fe through the iron-responsive element-iron responsive protein (IRE-IRP) pathway. Al^3+^ and/or Mn^3+^ can influence the IRE-IRP pathway. APP possesses Cu and/or Zn binding domains and reduces Cu^2+^ to Cu^+^. APP also regulates Fe^2+^ efflux by stabilizing ferroportin (FPN). AßP, which is derived from APP by the enzymatic cleavage of ß-secretase and γ-secretase. The primate AßP possesses the ability to bind metals such as Al, Zn, Cu, Fe, and Mn, although rodent AßP does not. The primary sequences of primate AßP and rodent AßP differ by only three metal-binding amino acids (Arg^5^, Tyr^10^, and His^13^; shown in Italic and Bold form). (**b**) Prion protein (PrP^C^). The expression of PrP is regulated by the level of Fe through the IRE-IRP pathway as well as APP. PrP^C^ can bind Cu^2+^, Zn^2+^, Mn^2+^, and other metal ions. Four Cu atoms can bind to the octarepeat domain in PrP^C^, and two Cu bind to other His residues (His^96^ and His^111^). PrP106-126, which is a neurotoxic fragment peptide, can bind to Cu and other metal ions. PrP^C^ possesses the ferrireductase activity that converts Fe^3+^ to Fe^2+^. (**c**) α-Synuclein. The expression of α-synuclein is regulated by the level of Fe through the IRE-IRP pathway as well as APP and PrP^C^. α-Synuclein reportedly binds Cu^2+^, Zn^2+^, Mn^2+^, and other metal ions in its N-terminal and C-terminal domains. α-Synuclein possesses the ferrireductase activity that converts Fe^3+^ to Fe^2+^, similar to PrP^C^.

AßP is a small 39–43-amino-acid peptide, which is derived from a large precursor protein (amyloid precursor protein: APP) by the enzymatic cleavage of ß-secretase in its N-terminal and γ-secretase in its C-terminal (Figure 2a). AßP(1–40), comprising the first 40 amino acids of AßP, was found to cause neurotoxicity in vitro as well as in vivo in 1991. After these findings were revealed, the conformational change of AßP was demonstrated to be a crucial determinant of its neurotoxicity [57]. AßP possesses a tendency to form insoluble aggregates (oligomers) with ß-pleated sheet structures in solution. Freshly prepared AßP(1–40), forming an α-helix or random-coiled structure in solution, is non-toxic. However, after an aging process (incubation at 37 °C for several days), the aged AßP(1–40) self-aggregates to form oligomers with ß-sheet structures and was found to cause the death of cultured rat cortical neurons. The proportion of ß-sheet content of AßP was shown to be correlated with its neurotoxicity. Mutations in APP have been revealed to be a risk factor for familiar-type AD. Presenilins (presenilin-1 and presenilin-2), risk factors of early-onset familial AD, are γ-secretases that influence the production of various truncated AßPs. Truncated AßPs, such as AßP(1–42), were revealed to be significant in oligomerization since they act as “seeds” and enhance the oligomerization of AßP(1–40). These biochemical and genetic findings support the “amyloid cascade hypothesis,” which proposes that the conformational changes of oligomerized AßP and the subsequent neurodegeneration are crucial for the pathogenesis of AD [58].

The levels of AßP in the CSF of the elderly without dementia are reportedly similar to those of AD patients, and AßP also exists in the CSF of young individuals [59]. Therefore, acceleratory or inhibitory factors of the oligomerization may be crucial in the pathogenesis of AD [57]. Substances such as rifampicin, transthyretin, curcumin, aspirin, and peptides (such as the ß-sheet breaker peptide) were reported to inhibit oligomerization and are now candidates for drugs to treat AD. The oligomerization of AßP is influenced by its concentration, the composition and pH of solvents, and temperature. The oxidation, mutation, and racemization of AßP accelerate the oligomerization. Among these acceleratory factors, trace elements are of particular interest. Metal ions bind to several amino acid residues, such as histidine (His), arginine (Arg), tyrosine (Tyr), glutamate (Glu), and aspartic acid (Asp) or phosphorylated amino acids. These metals cause conformational changes in various proteins by cross-link amino acid residues. Interestingly, the deposition of senile plaques rarely occurs in rodent brains compared with the level in primate brains, despite primate AßP and rodent AßP differing by only three amino acids (Arg^5^, Tyr^10^, and His^13^) [60]. These three amino acids bind to metal ions, and His residues are particularly essential for the binding of Zn to AßP. Therefore, metals may contribute significantly to the oligomerization of AßP and the deposition of senile plaques. Our and other numerous studies revealed that metal ions, including Al^3+^, Fe^3+^, Zn^2+^, Cu^2+^, and Mn^2+^, bind to AßP and enhance its oligomerization [61,62,63,64,65,66]. Moreover, the deposition of Al, Zn, Fe, and Cu was observed in senile plaques [67,68].

APP, the precursor of AßP, is also a metal-binding protein and possesses two Zn and/or Cu binding domains [69]. Both Zn and Cu are implicated in the dimerization of APP, its expression, trafficking, processing, and in the production of AßP [70,71]. APP has the ability to convert oxidized Cu^2+^ to reduced Cu^+^ [72]. Moreover, APP reportedly regulates Fe^2+^ efflux from neurons by stabilizing FPN [73]. Other AD-related proteins, presenilins, are involved in Ca homeostasis in the ER [74] and are implicated in the cellular uptake of Zn and Cu [75]. Meanwhile, Zn and Cu influence the trafficking of presenilins and their γ-secretase activity [76].

Fe regulates the expression of APP. The mRNA of APP contains an IRE domain in its 5′-UTR, similar to ferritin [77]. Therefore, the translation of APP is promoted in the case of excess Fe, and thereafter the export of Fe^2+^ by FPN can be upregulated to maintain intracellular Fe levels. Indeed, high-Fe diets were reported to cause the cleavage of APP and the accumulation of AßP in the brain, which was inhibited by intranasal exposure to deferoxamine, a chelator of Fe and Al [78].

Numerous other studies have suggested the involvement of metal homeostasis in AD. The genotype of TF C2 is a risk factor for AD [79]. Fe supplementation reportedly has beneficial effects on cognitive functions in AD patients [80]. Lu et al. demonstrated that the expression of DMT1 increases in an age-dependent manner, along with increases in AßP and phosphorylated tau [81]. The brains of AD patients exhibited a decreased level of FPN [82].

We summarize the links between trace elements and AßP/APP from the perspective of metal interactions in Figure 2a. Figure 2 also presents a comparison of other amyloidogenic proteins (PrP^C^ and α-synuclein).

### 3.2. Prion Diseases

Prion diseases are zoonotic diseases, including bovine spongiform encephalopathy (BSE) in cattle, scrapie in sheep, and chronic wasting disease in elk and deer. In humans, Creutzfeldt–Jakob disease (CJD), Gerstmann–Sträussler–Scheinker syndrome, and kuru are included [83]. They are characterized by the spongiform degeneration of glial cells and neurons and synaptic degeneration. The accumulation of abnormal scrapie-like prion protein (PrP^Sc^) is also observed in the brain. It is widely accepted that the conformational conversion of normal cellular prion protein (PrP^C^) to pathogenic PrP^Sc^ is central to the pathogenesis of prion diseases. PrP^C^ is a 30–35 kDa glycoprotein anchored at the plasma membrane with a glycosylphosphatidylinositol (GPI) domain. It is widely distributed throughout the body, including the liver, heart, and brain. PrP^C^ and PrP^Sc^ possess the same chemical characteristics with the same primary sequence, except that PrP^Sc^ is protease-resistant, insoluble, and has a high β-sheet content. Prion diseases are also called transmissible spongiform encephalopathy from their infective characteristics. PrP^Sc^ has the ability to induce neighboring normal PrP^C^ to misfold and aggregate PrP^Sc^. When PrP^Sc^ in food or iatrogenic contamination enters the body via ingestion, the protease-resistant PrP^Sc^ invades the brain and converts PrP^C^ to PrP^Sc^.

Metals are involved in both of the neurodegenerative processes in prion diseases: “loss of normal protective functions of PrP^C^” and “gain of toxic functions of PrP^Sc^.” According to the “loss-of-function neurodegenerative pathway,” several neurological dysfunctions, including the death of Purkinje neurons in the cerebellum, synaptic dysfunction, demyelination, depressive-like behavior, and memory loss in knockout mice of PrP^C^. In PrP-knockout mice, the decreased Cu levels and the reduced activity of Cu-dependent enzymes were demonstrated [84]. PrP^C^ possesses a highly conserved octarepeat domain composed of multiple tandem copies of an eight-residue sequence (PHGGGWGQ) (residues 66–90) at the N-terminal (Figure 2b). Jackson et al. reported that four Cu atoms bind to the His residues in the octarepeat domain of PrP^C^, as well as two other Cu atoms to other His residues (His^96^ and His^111^) [85]. They also demonstrated that other metals, including Zn^2+^, Mn^2+^, and Ni^2+^, bind to these binding sites with lower affinity than Cu^2+^. Additionally, PrP^C^ regulates Cu influx into neurons, and Cu-bound PrP^C^ exerts neuroprotective effects by exhibiting Cu/Zn SOD-like activity. Furthermore, PrP^C^ controls the functions of glutamate receptors (MPA-type and/or NMDA-type) in a Cu-dependent manner [86].

According to the “gain of toxic function neurodegenerative pathway,” a fragment peptide of PrP (PrP106–126) has been used as a model peptide of PrP^Sc^ because it causes the death of cultured hippocampal neurons, the ability to form fibrils with β-sheet structures, and the ability to bind Cu and Zn [87]. We have demonstrated that Zn and Cu attenuated the neurotoxicity of PrP106–126 and the formation of ß-sheet structures [88].

Cu also affects the cellular trafficking of PrP^C^ and its gene expression. Cu also influences PrP^C^-induced neurite outgrowth [89]. It is widely accepted that Cu contributes to the conformational conversion of PrP^C^ to PrP^Sc^ and the transmission of prion diseases [90]. It is known that the α-helix-rich C-terminal domain of PrP^C^ changes to the β-sheet in the conformational changes of PrP^Sc^. When Cu binds to the octarepeat region in the flexible N-terminal domain of PrP^C^, Cu crosslinks with His residues in the C-terminal domain and finally causes the conformational change of PrP^C^ to PrP^Sc^. Indeed, the mutation of ATP7A delays the onset of prion disease by disrupting Cu homeostasis in mice [91].

Zn also contributes to the conformational changes of PrP^C^ to PrP^Sc^. Zn^2+^ binds to the octarepeat domain as well as Cu^2+^ and affects the conformational changes of the C-terminal domain [92]. Bioinformatic analysis revealed that genes of PrP^C^ have evolutionary similarities to ZIP-type Zn transporters such as ZIP5, ZIP6, and ZIP10 [93]. Watt et al. reported that PrP^C^ acts as a Zn^2+^ sensor in the binding of synapses with the AMPA-type glutamate receptors and controls the cellular uptake of Zn^2+^ [94].

PrP^C^ is also involved in Fe homeostasis. Notably, PrP^C^ was revealed to be a ferrireductase that converts Fe^3+^ to Fe^2+^ [95]. The reduced Fe^2+^ is subsequently transported to intracellular space via the complex of DMT1 and ZIP14 and is provided to enzymes requiring bioactive Fe^2+^ [96]. The octarepeat domain was essential for this ferrireductase activity. Moreover, PrP-knockout mice exhibited altered Fe metabolism and reduced Fe levels in the brain [97]. Meanwhile, the Fe level controls the expression of PrP^C^ because its mRNA possesses an IRE motif as well as the APP gene [98].

Mn has been suggested to facilitate the pathogenesis of prion diseases [99]. Mn reportedly increases the infectivity of PrP^Sc^ in mice [100]. The risk of chronic wasting disease in deer was shown to be associated with Mg deficiency and increased Mn concentrations [101]. Scrapie-infected sheep exhibited elevated Mn levels in the blood and brain [102].

Trace elements are also involved in the pathogenesis of human prion diseases. CJD patients exhibited elevated levels of Mn in blood and brains [103]. The epidemiological studies suggested the relationship between the high incidence of CJD in Slovakia and the imbalance of the Mn/Cu ratio in food was suggested [104,105].

### 3.3. Lewy Body Diseases

The pathological hallmarks of LBD, which include Parkinson’s disease (PD), dementia with Lewy bodies (DLB), and multiple system atrophy, etc., are the abnormal intracellular aggregates termed “Lewy bodies.” The major component of Lewy bodies is α-synuclein [106]. Therefore, these diseases are also called “synucleinopathies.”

α-Synuclein is abundant in the presynaptic terminals, particularly in the membranes of synaptic vesicles. It plays central roles in endocytosis and exocytosis and contributes to synaptic plasticity. The fragment peptide α-synuclein termed the “non-amyloid component” (NAC), co-accumulates with AßP in senile plaques of AD [107]. The oligomerization of α-synuclein contributes to neurotoxicity and the pathogenesis of LBD.

PD patients exhibit damage in Fe-rich regions (such as the substantia nigra). Thus, the roles of trace elements in the pathogenesis of LBD have been suggested. Uverski et al. revealed that Al and other metal ions enhance the oligomerization of α-synuclein [108]. α-Synuclein reportedly binds Cu^2+^, Zn^2+^, Mn^2+^, and other metal ions in its N-terminal and C-terminal domains [109]. Cu affects the binding of α-synuclein to lipid membranes and its functions [110]. The binding of Cu to α-synuclein is supposed to be critical for the pathogenesis because the His^50^ residue, a mutation that is observed in familiar-type PD, is essential for Cu binding [111].

Interestingly, α-synuclein possesses ferrireductase activity that converts Fe^3+^ to Fe^2+^ as well as PrP^C^ [112]. This indicates that α-synuclein controls energy production and neurotransmitter synthesis in the presynaptic domain by providing bioavailable Fe^2+^ to enzymes requiring Fe^2+^ in the mitochondria or ER. This ferrireductase activity is related to Cu binding to α-synuclein. Indeed, the levels of Fe^3+^ were altered in PD [113,114], and the ferrireductase activity was reduced in PD patients [115].

The expression of α-synuclein is controlled by Fe levels because its mRNA possesses an IRE motif in the 5′-UTR similar to APP and PrP^C^ [116]. As noted, the neurotoxicity of Mn shows a similar profile to PD. Mn reportedly induces the overexpression of α-synuclein by affecting the IRE-IRP pathway [117].

### 3.4. Vascular-Type Senile Dementia

VD is a type of senile dementia that accounts for approximately one-third of the cases. VD is generally caused by a series of small strokes or ischemia [118]. Within three months of the initial stroke, around 30% of the patients exhibit symptoms of dementia. During the interruption of blood flow in transient global ischemia or stroke, the resulting oxygen–glucose deprivation causes long-lasting membrane depolarization of neurons. Hereafter, the excess glutamate is released into the synaptic clefts, which induces overstimulation of glutamate receptors. Finally, the resulting Ca^2+^ influx triggers delayed neuronal death in the hippocampus or cerebral cortex, which are critical for memory formation.

Under ischemic conditions, excess Zn is secreted into synaptic clefts along with glutamate. The Zn translocation, namely, the increase in intracellular Zn^2+^ levels in postsynaptic neurons, reportedly occurs in degenerated hippocampal neurons after ischemia [119]. This Zn translocation is controlled by VDCC, NMDA-type glutamate receptor, and Ca^2+^-permeable AMPA-type glutamate receptor [8]. The administration of Ca-EDTA, a membrane-impermeable chelator of Zn^2+^, inhibits the translocation of Zn after transient global ischemia, protects hippocampal neurons, and reduces infarct volume [120]. These results strongly suggest that Zn is a key player in delayed neuronal death after transient global ischemia and, finally, in the pathogenesis of VD [121]. We have investigated the molecular mechanism of Zn-induced neurotoxicity and found associations with the ER stress pathway, mitochondrial energy failure, the disruption of Ca homeostasis, the MAP kinase pathway (stress-activated protein kinases/c-Jun amino-terminal kinase (SAPK/JNK) pathway), and the production of ROS [4]. We also found that sublethal concentrations of Cu^2+^ significantly exacerbated Zn-induced neurotoxicity [122] and demonstrated that the addition of Cu^2+^ produced ROS, which is known to induce the ER stress pathway and the SAPK/JNK pathway. Several antioxidants were found to attenuate Cu-enhanced Zn-induced neurotoxicity. Therefore, Cu may collaborate with Zn and play key roles in Zn-induced neurotoxicity by producing ROS, finally leading to the pathogenesis of VD [123].

## 4. Hypothesis: Cross-Talk Occurs between Trace Elements and Amyloidogenic Proteins at Synapses

As described in the previous sections, amyloidogenic proteins share several common characteristics, including the formation of ß-pleated sheet structures, the exhibition of neurotoxicity, and the ability to bind metals. Furthermore, all of these amyloidogenic proteins or related proteins (APP and PSs) reportedly co-localize at synapses. APP primarily exists in the presynaptic membrane [124], while PrP^C^ is located in the postsynaptic membrane, binding to several receptors [125]. α-Synuclein mainly exists in presynaptic cytosol or membranes, particularly in the presynaptic vesicles [106]. PSs are mainly localized in the ER membranes of pre and postsynaptic domains [126]. Considering that synaptic degeneration (synaptotoxicity) occurs in the early stages of these neurodegenerative diseases [127], the roles of amyloidogenic proteins at synapses are of interest.

**Figure 3:**
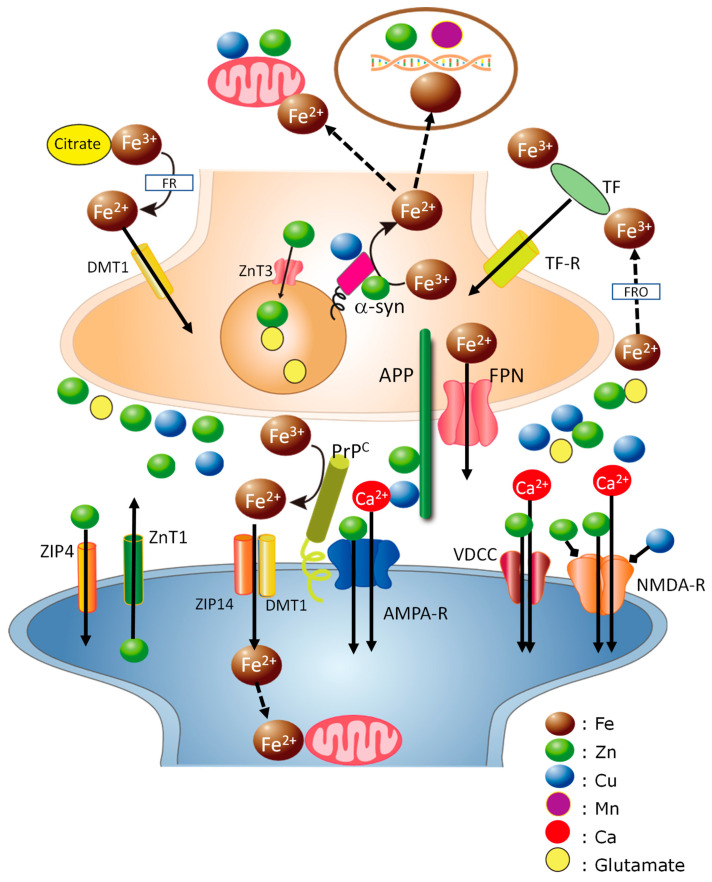
Hypothetical scheme of the roles of trace elements in synapses: Overview of the entry of trace elements and its roles in synapses. APP is primarily localized to the presynaptic membrane. PrP^C^ is localized to the postsynaptic membrane. α-Synuclein is mainly localized to the presynaptic domain and membranes of synaptic vesicles. Orally administered Fe is absorbed in the gastrointestinal tract and transported in the blood as Fe^3+^ by binding to transferrin (TF) or citrate. TF-bound Fe^3+^ can enter neurons or glial cells via its receptors (TF-R). Thereafter, Fe^3+^ is converted to Fe^2+^ by ferrireductases (FR). α-Synuclein acts as a ferrireductase in the presynaptic domain and functions to provide bioavailable Fe^2+^ to enzymes requiring it in mitochondria and other organelles. PrP^C^ acts as a ferrireductase in the postsynaptic domain, and the converted Fe^2+^ is transported into cells by the complex of ZIP14 and divalent metal transporter 1 (DMT1). Fe^3+^ binding with citrate is also reduced to Fe^2+^ by FR and enters cells via DMT1. Excess intracellular Fe^2+^ is exported via ferroportin (FPN). APP stabilizes FPN and regulates Fe^2+^ efflux from cells. α-Synuclein and FPN coexist in presynaptic vesicles and control Fe homeostasis in the vesicles. Excess synaptic Fe^2+^ is oxidized to Fe^3+^ by ferroxidase (FRO) and turns into circulation. Meanwhile, Fe controls the expressions of these proteins. Mn can influence this IRE-IRP pathway and affect the expressions. Orally administered Zn and Cu enter into the neurons or glial cells, accumulate in synaptic vesicles, and are activity-dependently released to the synaptic clefts. Secreted Zn as well as Cu bind to NMDA-type glutamate receptors or other receptors, and control neuronal excitability. Synaptic Zn is translocated into postsynaptic neurons through voltage-dependent Ca^2+^ channels (VDCC), NMDA-type glutamate receptor, and Ca^2+^-permeable APMA receptor as well as Ca^2+^. PrP^C^ acts as a Zn sensor and transports Zn to postsynaptic neurons. Zn transporters such as ZnT1 and ZIP4 contribute to regulating Zn levels in the synaptic clefts. NMDA-R, NMDA-type glutamate receptor; AMPA-R, AMPA-type glutamate receptor; FPN, ferroportin; TF, transferrin; VDCC; voltage-dependent calcium channel, colored circles represent Zn, Cu, Fe, Mn, Ca, and glutamate.

**Figure 4:**
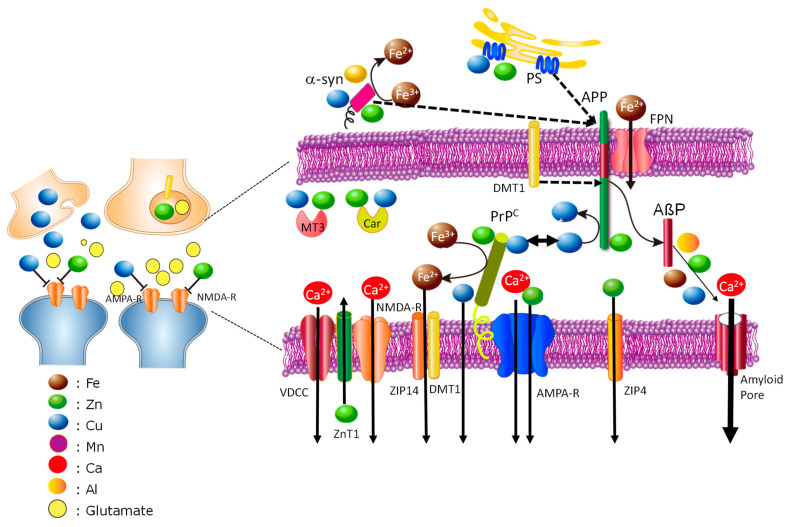
Hypothetical scheme of the roles of trace elements in synapses: Detailed cross-talk of trace elements and amyloidogenic proteins at synaptic clefts. Zn and Cu are secreted to the synaptic clefts during neuronal excitation and transmit the information of neuronal firing to neighboring synapses by binding to NMDA-type glutamate receptors and other receptors. It is suggested that both Zn and Cu contribute to synaptic plasticity. In postsynaptic membranes, PrP^C^ binds to Cu, Zn, and Fe. PrP^C^ acts as a Zn^2+^ sensor and contributes to the uptake of Zn by AMPA-type glutamate receptors. Additionally, PrP^C^ acts as a ferrireductase and controls Fe influx via ZIP14/DMT1. Other Zn transporters (ZnT1 and ZIP4) also localize at the postsynaptic domain and maintain Zn^2+^ levels in the synaptic clefts. ZnT1 also regulates NMDA-R and Ca^2+^ channels. APP binds to Cu and/or Zn in the presynaptic membranes. Considering the narrowness of the synaptic clefts (approximately 20 nm), PrP^C^ may provide Cu^2+^ to APP, and APP converts Cu^2+^ to Cu^+^ and may provide Cu^+^ to copper transporter 1 (CTR1) for re-uptake into presynaptic vesicles. Zn^2+^ and Cu^2+^ influence the secretion of AβP from APP by binding with presenilins (PS), and γ-secretases. Metallotionein3 (MT3) and carnosine (Car) also exist in synaptic clefts and regulate the homeostasis of Zn and Cu. In pathological conditions, AβP and other amyloidogenic proteins form pore-like structures on membranes. The amyloid pores cause Ca^2+^ dyshomeostasis and trigger various neurodegenerative processes. Trace elements can contribute to the formation of amyloid pores by accelerating the oligomerization of amyloidogenic proteins. NMDA-R, NMDA-type glutamate receptor; AMPA-R, AMPA-type glutamate receptor; FPN, ferroportin; PS, presenilins; VDCC, voltage-dependent calcium channel; colored circles represent Fe, Zn, Cu, Fe, Mn, Al, and glutamate.

Synapses are metal-rich regions of the brain. The levels of Cu and Zn in the CSF of healthy individuals are less than 1 µM [128]. However, Cu and Zn may be concentrated in the synaptic clefts. The synaptic cleft is a small compartment considered to be a cylinder with a 120 nm radius and height of 20 nm. The total volume of synaptic clefts is estimated to be approximately 1% of the extracellular space of the brain [129]. Vogt et al. estimated that the Zn concentration in synapses is 1–100 μM [130]. Meanwhile, the concentration of Cu released into the synaptic cleft is estimated to be 3–100 μM [131,132]. Considering that synapses are energy-demanding regions in the brain [133] and that Fe is required in energy metabolism in mitochondria, Fe may plausibly be abundant in synapses. Considering these results together, we hypothesize here that amyloidogenic proteins and metals may play concerted roles in maintaining metal homeostasis in the synapse (Figure 3 and Figure 4).

Figure 3 presents overall aspects of the entry of trace elements into neurons and their functions in pre and postsynapses. Orally administered Fe is absorbed via the gastrointestinal tract and transported in the blood by binding to TF or citrate. TF-bound Fe passes the BBB and enters neurons or glial cells via the TF receptor. Intracellular Fe^3+^ is converted to Fe^2+^ by α-synuclein in the presynaptic domain and by PrP^C^ in the postsynaptic domain; then, the bioavailable Fe^2+^ is provided to enzymes that require it in mitochondria or other suborganelles and functions in neurotransmitter synthesis, energy production, and myelination. Fe^3+^ in NTBI enters neurons or glial cells via DMT1 after being reduced to Fe^2+^. Excess Fe^2+^ is exported to extracellular space via FPN and then oxidized to Fe^3+^ by ferroxidase to enter into circulation. APP stabilizes FPN and plays a key role in Fe export. FPN is reportedly localized in membranes of synaptic vesicles as well as α-synuclein [134] and modulates Fe homeostasis. Since synapses are energy-demanding areas and therefore vulnerable to oxidative stress, the Fe^2+^ levels there should be strictly regulated by these concerted actions of APP, PrP^C^, and α-synuclein. Meanwhile, Fe controls the expression of APP, PrP^C^, and α-synuclein, given that their mRNA possesses the IRE motif in the 5′-UTR. Moreover, DMT1 is involved in the processing of APP and the production of AßP [135].

Both Zn and Cu are absorbed via the gastrointestinal tract, transported in the blood flow, pass through the BBB, and enter neurons or glial cells. Considerable amounts of Zn and Cu accumulate in synaptic vesicles, both of which are released to the synaptic clefts in activity-dependent manner, bind to NMDA-type glutamate receptors or other receptors, and control neuronal excitability under physiological conditions. Synaptic Zn and Cu may play inhibitory roles in translating the information about neuronal firings to neighboring synapses and may contribute to synaptic plasticity. Secreted Zn is translocated by VDCC, NMDA-type glutamate channels, and Ca^2+^-permeable AMPA-type glutamate channels, as well as Ca^2+^. Moreover, PrP^C^ acts as a Zn sensor in the synaptic clefts and regulates Zn influx to postsynaptic neurons with AMPA-type glutamate receptors as a ZIP analog. PrP^C^ also plays a key role in the uptake of Cu and Fe. Two major Zn transporters—ZnT1 and ZIP4—are also present in the postsynaptic domain and act to maintain the level of Zn in the synaptic clefts [136,137].

More details on the interactions in the synaptic clefts are shown in Figure 4. As noted, the levels of Zn, Cu, and Fe in the synaptic clefts are strictly regulated by the interactions between amyloidogenic proteins.

The height of the synaptic clefts, namely, the distance from presynaptic membranes to postsynaptic membranes, is as narrow as 20 nm, and therefore these amyloidogenic proteins possibly interact with each other. Indeed, PrP^C^ acts as a toxic receptor of AßP and contributes to its toxicity [138]. α-Synuclein increases AßP secretion by promoting the processing of APP by β/γ-secretase [139]. Presenilins also regulate the secretion of APP by binding to Zn and/or Cu [76]. As noted, ZnT1 and ZIP4 control the level of Zn in the synaptic clefts. ZnT1 also regulates neuronal excitability by binding to NMDA-type glutamate receptors [136] and controls Ca homeostasis by binding to Ca^2+^ channels [140].

Other factors, such as MT3 and carnosine (ß-alanyl histidine), also control the levels of Zn and Cu in the synapses. MT3, brain-specific metallothionein, is synthesized in neurons or glial cells [141]. Interestingly, the brains of AD patients exhibited a decreased level of MT3. Carnosine is an endogenous dipeptide with various beneficial properties, such as antioxidant, anti-glycation, anti-stress, and anti-cross-link. Carnosine also has the ability to scavenge hydroxyl radicals, maintain the pH balance, and chelate metals, including Zn^2+^ and Cu^2+^ ions [142]. Carnosine is reportedly synthesized in oligodendrocytes and secreted into the synaptic cleft [143].

These concerted metal–protein, and metal–metal interactions at synapses are highly complex and vulnerable to perturbation. Therefore, the disorder of these subtle interactions may cause the dyshomeostasis of neurometals, ROS production, and synaptic degeneration, and finally induce neurodegeneration leading to the pathogenesis of the neurodegenerative disease.

For example, high-Fe diets reportedly cause the cleavage of APP and the accumulation of AßP in the brain [78]. The abnormality of APP induced by its mutations may influence FPN stability and the export of Fe^2+^. A decreased level of FPN, which is reported in AD brains [82], causes the accumulation of Fe^2+^ and the production of ROS. The invasion of Al from food enters the neurons via TF receptors as well as Fe and influences various Fe-related pathways, such as the IRE-IRP pathway, and thus influences the expression of APP [144]. Al reportedly enhances the expression of APP and the production of AßP [145]. Dietary Cu and its oxidative stress have been linked with AD pathogenesis [146]. Excess Mn from environmental exposure influences the IRE-IRP pathway and causes the accumulation of Fe and the overexpression of α-synuclein [49,117].

Contaminating PrP^Sc^ from food or an iatrogenic device, which is resistant to digestion by proteases, passes into the brain, causes the conversion of normal PrP^C^ to PrP^Sc^, leads to the loss of neuroprotective functions of PrP^C^, and triggers neurotoxic pathways via the accumulation of metals. Mutations of α-synuclein influence its binding to Cu, which affects the membrane binding and ferrireductase activity, causes changes in the Fe^2+^/Fe^3+^ ratio, and finally leads to ROS production.

The Cu and Zn levels in the synapses are strictly controlled by several regulatory factors, and secreted Zn and Cu rapidly undergo re-uptake under physiological conditions. However, under pathological conditions such as transient global ischemia, sustained excitation of neurons occurs for prolonged periods in broad areas of the brain, and excess Zn and Cu are simultaneously secreted into the same synaptic clefts for a long period of time. We have demonstrated that a sublethal concentration of Cu (5 μM) significantly exaggerated 30 μM Zn-induced neurotoxicity (molar ratio of Zn:Cu = 6:1) [123]. Therefore, it is possible that the coexistence of Cu triggers the production of ROS and cooperatively enhances Zn-induced neurotoxicity, finally causing vascular-type dementia. Cu has been found to be a risk factor for stroke in several epidemiological studies [147,148].

Trace elements possibly affect the gain of toxic functions of amyloidogenic proteins. Researchers, including ourselves, have demonstrated that AßP forms pore-like structures on the membrane and causes Ca dyshomeostasis, which may be associated with neurotoxicity [149,150,151,152]. PrP106–126 also forms cation-permeable pores in a Cu-sensitive manner as well as AβP [153]. α-Synuclein reportedly forms annular pore-like structures similar to AβP(1–40) [154]. Trace elements can influence neurotoxicity by accelerating the oligomerization and the formation of amyloid pores. However, the roles of metal-induced oligomerization are still controversial and under investigation. The morphologies of AβP oligomers with Al, Cu, Fe, and Zn are quite different [155]. The effects of metal-induced oligomers on the neurotoxicity of AβP are not identical [156,157].

On the basis of this working hypothesis, a metal-based therapeutic pathway may become a good candidate for the treatment/prevention of these neurodegenerative diseases. Clioquinol, a chelator of Zn and Cu, and its analog PBT-2 have been examined for possible treatment of AD [158], although the results remain controversial [159]. Exley et al. demonstrated the efficacy of silicate, which chelates Al, as a possible treatment of AD [160]. Deferoxamine (DFO), a chelator of Fe and Al, exerts beneficial effects on maintaining the daily living skills of AD patients [161,162]. Recent approaches using intranasal DFO treatment attenuated synapse loss in the brains of AD model mice and inhibited Fe-induced APP cleavage [78,163]. DFO and other Fe chelators are focused on possible treatments for PD [164,165]. Moreover, clioquinol reportedly recovers the memory impairment in scrapie [166], and a Cu^2+^-specific chelator (D-(-)-penicillamine) reportedly inhibits the pathogenesis of prion diseases in vivo [167]. We focused on carnosine as a possible treatment for VD based on its neuroprotective effect against Zn-induced neurotoxicity [168] and its various beneficial characteristics [142]. Carnosine also reportedly inhibits the oligomerization of AβP and attenuates neurodegeneration in AD model mice [169], and protects against ischemia-induced neuronal death in vivo [170]. Carnosine also attenuates PrP106–126-induced neurotoxicity [88]. Based on these beneficial characteristics of carnosine as a neuroprotector, we have granted a patent for carnosine as a possible target for drug treatment of vascular-type senile dementia [171].

## 5. Conclusions

We have reviewed the enigmatic roles of trace elements in the pathogenesis of various neurodegenerative diseases based on common characteristics of amyloidogenic proteins and the cross-talk between metal ions at synapses. Our hypothesis about the common aspects of neurodegenerative processes may shed light on their pathogenesis. Based on the idea about the link between trace elements and the pathogenesis of neurodegenerative disease will lead to the development of drugs for these neurodegenerative diseases.

However, this hypothesis is still not completed. In particular, the levels of Zn, Cu, and Fe in the synapses are controversial and under investigation. The functions of amyloidogenic proteins and the localizations of metal transporters remain unsolved. However, since we published the prototype of this hypothesis in 2017 [172], great progress has been demonstrated in this research field, particularly in the functions of Fe and in localizations of metal transporters. We hope that future studies about the metals in synapses will evolve the hypothesis and will aid in the treatments for these neurodegenerative diseases.
